# Hypertension, an Emerging Problem in Rural Cameroon: Prevalence, Risk Factors, and Control

**DOI:** 10.1155/2016/5639146

**Published:** 2016-12-08

**Authors:** Walters Tabi Arrey, Christian Akem Dimala, Julius Atashili, Josephine Mbuagbaw, Gottlieb Lobe Monekosso

**Affiliations:** ^1^Ako District Hospital, North West Region, Cameroon; ^2^Faculty of Epidemiology and Population Health, London School of Hygiene and Tropical Medicine, London, UK; ^3^Orthopaedics Department, Southend University Hospital, Essex, UK; ^4^Health and Human Development (2HD) Research Group, Douala, Cameroon; ^5^Faculty of Health Sciences, University of Buea, Buea, Cameroon; ^6^College of Medicine, Madonna University Nigeria, Rivers State, Nigeria

## Abstract

*Background*. Despite the increasing trends suggesting that hypertension is a growing public health problem in developing countries, studies on its prevalence, associated risk factors, and extent of blood pressure control have been inequitably done in urban and rural communities in these countries. We therefore aimed to determine the prevalence of hypertension and extent of blood pressure control in rural Cameroon.* Methods*. This was a community-based cross-sectional study conducted in rural Cameroon (the Moliwe Health Area). Participants aged 21 years and above were recruited by a probability proportional to size multistage sampling method, using systematic sampling for household selection and random sampling for participant selection. Blood pressure, weight, and height were measured by standard methods. Hypertension was defined as BP ≥ 140/90 mmHg.* Results*. The prevalence of hypertension among the 733 participants recruited was 31.1% (95% CI: 27.8–34.6) and 71% (95% CI: 58.7–81.7) of these hypertensive patients were newly diagnosed. Only 21.2% (95% CI: 12.1–33.3) of known hypertensives had a well controlled BP. Age, obesity, low educational status, and being married were associated with HTN after adjusting for confounders.* Conclusions*. The high prevalence of hypertension and inadequate BP control among known hypertensives in rural Cameroon warrants greater sensitization and regular screening to reduce hypertension-related morbidity and mortality.

## 1. Background

It is predicted that by 2025, the prevalence of hypertension (HTN) will increase by 60% to a total of 1.56 billion worldwide [1] suggesting that HTN remains a major public health problem. HTN in Sub-Saharan Africa (SSA) has also been on the rise with reports indicating higher values in urban settings compared to rural settings [2–4]. The prevalence of HTN in SSA ranges between 14.5% in rural Eritrea [2], 32.9% in semiurban Ghana [3], and 40.1% in urban South Africa [4]. Likewise, adequate blood pressure (BP) control has been on the decline, ranging between 1.7% in rural Ghana [3], 4% in urban slum dwellers in Nigeria [5], and 21.5% in urban Kenya [6]. In Cameroon, the prevalence of HTN spans from 5.7% in rural settings [7] through 21.9% in semiurban [8] to 47.5% in urban milieu [9], with a national average survey of 31.0% [10]. Despite the relatively better BP control in urban over rural settings, levels of adequate BP control as low as 2% [11] to 27.5% (in men) and 38.7% (in women) [9] have been reported in urban settings.

Even though much has been done so far to reduce the incidence of hypertension in urban areas, this has not been the same in rural areas, with public health policies aimed at controlling hypertension mainly directed towards the large cities. Most of the studies done so far in these urban areas cannot therefore give a true estimate of the extent to which the rural population is affected by this major cardiovascular disease risk factor. An analysis of hypertension and its risk factors in rural Cameroon will therefore help guide health policy decisions and provide baseline data for future studies aimed at addressing this problem. We therefore had the following as objectives: determining the prevalence of hypertension in the adult population in the Moliwe Health Area in rural Cameroon; identifying the risk factors associated with HTN in this rural setting; determining the extent of BP control in known hypertensive patients in this locality.

## 2. Materials and Methods

### 2.1. Study Design, Period, and Setting

A community-based cross-sectional descriptive and analytical study was conducted over a 6-month period (June 20 to December 28, 2013) in the Moliwe Health Area (MHA). This principally rural health area is found in the Limbe Health District of the South West Region of Cameroon.

### 2.2. Participants and Sampling Method

A multistage sampling method was used to select participants from the 5 villages/settlements of the health area: Bonadikombo, Wotutu, Ewongo, Moliwe, and Tomatal. The sample selected was self-weighted through the probability proportional to size method used. The data of 2005 Cameroon census was used as a guide to the sampling frame with households considered as the primary sampling units. Using a random start, a systematic sample of households was then selected from a list of the approximate cumulative number of households of all the villages. This gave an estimate of the number of households to be selected from each village. As the secondary sampling units, two participants were then recruited from each selected household by simple random sampling from a list of eligible households occupants produced by the respective household heads. Three hundred and twenty-nine participants were thus recruited from Bonadikombo, 113 from Wotutu, 112 from Moliwe, 96 from Ewongo, and 83 from Tomatal.

The sample size was calculated using the formula: *n* = *z* ^2^(*p*)(*q*)/*d* ^2^. A combined estimate of the prevalence of HTN in rural Rwanda, Tanzania, and Malawi of 22% was used [12]. We recruited 733 participants by systematic and simple random sampling methods.

### 2.3. Study Procedures and Variables

At each selected household, a questionnaire was administered to each recruited participant to collect information on age, sex, level of education, marital status, status of HTN, treatment of HTN, smoking, quantity of alcohol intake, physical activity, diabetes status, and family history of HTN. Blood pressure measurements were also done at the home of the participants by a casually dressed clinician not in white coat. Two measurements (in mmHg) were taken on the right arm using an automated electronic BP machine (OMRON M3 HEM-7200-E Omron Matsusaka Co. Ltd., Kyoto, Japan) through the standard procedure (JNC 7 recommendations) [13]. Heights in meters (m) and weights in kilograms (kg) were measured using standard procedures and were used to determine the body mass index (BMI) as follows: BMI = weight (kg)/[height (m) *∗* height (m)]. WHO STEPS surveillance manual was used to assess sedentary lifestyle (physical inactivity) [14].

### 2.4. Data Analysis

The data was entered into Epi info statistical software, version 7.0 (CDC/WHO, Atlanta, USA). Quality control was done by double entry and checking. Frequencies and means were obtained for appropriate variables; Chi-square and Fischer's exact tests were used as appropriate to test for associations between binary and categorical variables. A logistic regression model was built to assess the effect of factors found to be significantly associated with hypertension while controlling for potential confounders. Statistical significance was considered at a *P* value < 0.05.

### 2.5. Ethical Considerations

An ethical clearance was obtained from the Institutional Review Board of the Faculty of Health Sciences, University of Buea. Administrative approvals were obtained from the Regional Delegate of Public Health as well as from the local Chiefs, Quarter heads, and camp presidents concerned. There were no associated risks since all procedures were noninvasive. Participants benefited from free screening for hypertension and counselling. All patients found to have elevated blood pressure values were advised and referred to the nearest health facility for proper management and follow-up.

## 3. Results and Discussion

### 3.1. Sociodemographic and Clinical Characteristics of the Study Population

Of the 733 participants, 479 (39.5%) were between 20 and 29 years. The male to female ratio was 1 : 1.2, 35.9% had primary education, and 50.5% were married. Less than half of the participants (47.3%) had normal BMI and 15% had sedentary life style. A small proportion, (2.9%) were known diabetics and 32.7% reported a family history of hypertension (Table 1).

### 3.2. Prevalence of Hypertension and BP Control

**Figure 1:**
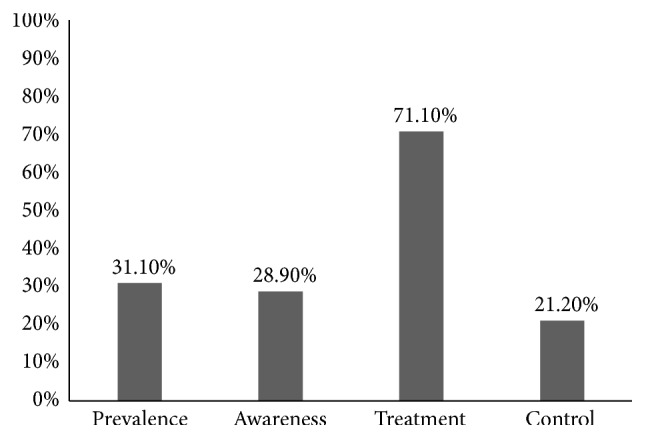
Descriptive characteristics of hypertension for the 733 participants in the Moliwe Health Area, Cameroon.

Of the 733 participants recruited, 228 were hypertensive, giving a prevalence of 31.1% (95% CI: 27.8–34.6). Sixty-six of the 228 hypertensive participants (28.9%, 95% CI: 23.2–35.3) knew their status prior to our study. Of these 66 known hypertensive participants already aware of their status, 14 of them (21.2%, 95% CI: 12.1–33.3) had their blood pressures well controlled even though as high as 47 of them (71%, 95% CI: 58.7–81.7) were on antihypertensive treatment at that moment (Figure 1).

Age 40 and above, obesity, smoking, alcohol consumption, diabetes, low educational level, and marriage were found to be associated with hypertension (Table 2). After adjusting for all significant factors using logistic regression, only age 40 and above, obesity, low educational level, and marriage were still strongly associated with hypertension (Table 3). There was a progressive increase in the prevalence of HTN with age when age below 40 was used as the reference: from 3 folds in the 40–59 years, through 8.3 folds in the 60–79 years, to 11.6 folds in 80^+^ years age groups. People who were obese had 2.8 times the odds of being hypertensive compared to the nonobese. There was an inverse relationship between educational status and HTN. Those who never went to school had 6.7 times the odds of having HTN compared to those who had at least high school education (Table 3).

### 3.3. Discussion

Since the new recommendations for HTN diagnosis and management by JNC 7 in 2003 [13], no study had been done in rural Cameroon. The last published rural study on HTN was as far back as 1998 [7]. The 31.1% prevalence of HTN observed in the MHA is similar to the 31.0% reported in Cameroon by Kingue et al. [10] and the 32.9% reported by Amoah [15] in semiurban Ghana but higher than that reported elsewhere [3, 7, 8, 12, 16, 17]. The different settings and methodologies could account for this difference. However, our value was lower than 44.7% reported by Williams et al. [18] in rural Ghana and 40% reported by Chow et al. [19] in rural and urban high, middle, and low income countries.

Our study revealed low hypertension awareness similar to the 32.3% observed in rural Ghana by Addo et al. [20] and the 32.6% observed by Dzudie et al. [9] in Urban Cameroon. However, this value is higher than the 11% reported in rural Cameroon by Mbanya et al. [7] and 22% in rural and semiurban Ghana by Williams et al. [18]. This variation between rural and urban settings could be explained by poor knowledge of the disease, lesser access to health facilities, and poverty, in rural compared to urban communities.

Age was identified to be strongly associated with HTN, supporting the findings of other studies [9, 12, 21–23]. This is because as people get older, their blood vessels become harder and they are likely to have decreased baroreceptor sensitivity, increased responsiveness to sympathetic nervous system stimuli, altered renal and sodium metabolism, and an altered renin-aldosterone relationship thereby predisposing them to high blood pressure [24]. Obesity, which is one of the common pathways between diabetes and HTN [25], was also found to be strongly associated with HTN in our study, as earlier documented [8, 26]. Low educational status and being married were strongly linked with HTN in our study as reported in previous studies [21, 27, 28]. A poor understanding of the disease and its risk factors and the stress experienced my married couples, respectively, could put these groups at a higher risk. On the other hand, gender, family history of HTN, overweight, and physical inactivity were not statistically significant risk factors of HTN in this setting. Smoking, alcohol consumption, and diabetes mellitus which were associated with HTN on bivariate analysis were not significantly associated with hypertension on multivariate analysis by logistic regression.

The proportion of known hypertensives who were currently on antihypertensive treatment in our study was similar to the 64.9% reported [18] in Ghana but much higher than the 12.5% reported [7] in Cameroon. The high proportion on antihypertensives may be due to the increase in awareness of HTN and the availability and access to antihypertensive medications in Cameroon lately compared to 16 years ago [7]. Despite this considerable proportion of hypertensive patients being on treatment, only 21.8% of them had controlled blood pressures. Kishore et al. reported as low as 3% in rural Nigeria [23] and Yuvaraj et al. reported 12.5% in rural India [29]. However, higher proportions have been reported (33.1% [9] and 63.2% [8]) among hypertensives in urban and semiurban settings in Cameroon, respectively, 56.8% in Mexico [30], and 75.9% in urban India [31]. Noncompliance to treatment has been proposed as a possible explanation for this observation. And in this context noncompliance to treatment is multifactorial in aetiology with reasons such as inadequate knowledge of HTN, intolerance of side effects of medications, and low purchasing power.

This study was cross-sectional which means participants declared hypertensive may not necessarily be hypertensive clinically. Also, our study was done in a small rural area whose findings may not necessarily apply to all rural Cameroonian or Sub-Saharan African communities. Moreover, some known risk factors such as dyslipidaemia, salt intake, and psychosocial and socioeconomic status were not assessed in our study. Nevertheless, the large sample size, varied sampling method, and execution of standard measurement procedures make the findings of this study robust and accurate. Also, the white coat effect which could potentially produce an overestimation of the actual prevalence of hypertension in this setting was minimized since the casually dressed clinician without a white coat did the measurements from the participants' homes.

## 4. Conclusions

Our study revealed that about one out of three adults in the Moliwe Health Area could be hypertensive with only a quarter of them being aware of their status and a fifth of those on treatment having their blood pressure well controlled. This reveals how very much a cardiovascular risk the rural population may be exposed to. There is, therefore, need for massive improvement in awareness through education and repeated patient follow-up in these rural settings alongside the urban ones. Also, the importance of further research in other rural communities to assess trends and risk factors of hypertension and the extent of blood pressure control cannot be overemphasized.

## Figures and Tables

**Table 1:** Participants' socio-demographic and clinical characteristics.

Characteristic	Categories	Participants (*N* = 733)	Percentage (%)
*Age (years)*	20–39	479	65.4
	40–59	167	22.8
	60–79	78	10.6
	80–99	9	1.2
*Gender*	Males	334	54.4
	Females	399	45.6
*Level of education*	None	74	10.1
	Primary	263	35.8
	Secondary	246	33.6
	High school or more	150	20.5
*Marital status*	Married	370	50.5
	Single	283	38.6
	Divorced	15	2.0
	Widowed	65	8.9
*Body mass index*	Normal	347	47.3
	Overweight	224	30.6
	Obese	162	22.1
*Smoking*	Yes	149	20.3
	No	584	79.7
*Alcohol consumption*	Yes	281	38.3
	No	452	61.7
*Sedentary lifestyle*	Yes	108	14.7
	No	625	85.3
*Family history of HTN*	Yes	240	32.7
	No	493	67.3
*Diabetes mellitus*	Yes	21	2.9
	No	722	97.1

**Table 2:** Association between clinical characteristics and hypertension of 733 participants in Moliwe Health Area, rural Cameroon.

Risk factors	Categories	Risk ratio (95% CI)	*P* value
*Age (years) *	<40	1.0	<0.001
	≥40	4.5 (3.0–6.8)	
*Obesity*	No	1.0	<0.001
	Yes	1.9 (1.5–2.3)	
*Smoking*	No	1.0	<0.001
	Yes	1.5 (1.2–1.9)	
*Alcohol*	No	1.0	<0.001
	Yes	1.6 (1.3–1.9)	
*Diabetes*	No	1.0	<0.001
	Yes	2.1 (1.4–2.9)	
*Educational status*	High (≥secondary)	1.0	<0.001
	Low (none and primary)	1.8 (1.4–2.3)	
*Marital status*	Unmarried	1.0	<0.001
	Married	1.6	

**Table 3:** Association between clinical characteristics and hypertension after adjusting for all the factors presented.

Risk factors	Categories	Odds ratio (95% CI)	*P* value
*Age (years) *	20–39	1.0	
	40–59	3.0 (2.0–4.6)	<0.001
	60–79	8.3 (4.4–15.7)	<0.001
	80 and above	11.6 (2.1–64.6)	<0.001
*Educational status*	High	1.0	
	None	6.7 (3.6–12.4)	<0.001
	Primary	2.0 (1.3–3.2)	<0.001
	Secondary	1.2 (0.7–2.0)	0.440
*Body mass index*	Normal	1.0	
	Overweight	1.0 (0.7–1.5)	0.850
	Obese	2.8 (1.9–4.2)	<0.001
*Smoking*	No	1.0	
	Yes	1.3 (0.8–2.0)	0.330
*Alcohol*	No	1.0	
	Yes	1.4 (0.9–2.1)	0.070
*Diabetes*	No	1.0	
	Yes	1.4 (0.5–3.9)	0.550
*Marital status*	Unmarried	1.0	
	Married	1.5 (1.1–2.2)	0.030

## Abbreviations

BP: Blood pressure
CDC: Centre for Disease Control and Prevention
CVD: Cardiovascular disease
HTN: Hypertension
JNC: Joint National Committee
MHA: Moliwe Health Area
SSA: Sub-Saharan Africa
USA: United States of America
WHO: World Health Organization.

## References

[1] Kearney P. M., Whelton M., Reynolds K., Muntner P., Whelton P. K., He J. (2005). Global burden of hypertension: analysis of worldwide data. *The Lancet*.

[2] Mufunda J., Mebrahtu G., Usman A. (2006). The prevalence of hypertension and its relationship with obesity: results from a national blood pressure survey in Eritrea. *Journal of Human Hypertension*.

[3] Cappuccio F. P., Micah F. B., Emmett L. (2004). Prevalence, detection, management, and control of hypertension in Ashanti, West Africa. *Hypertension*.

[4] Malhotra R., Puone T., Hoyo C., Hughes G. (2008). Prevalence and awareness of hypertension in an urban township of South Africa: compelling need for action. *Ethnicity and Disease*.

[5] Daniel O. J., Adejumo E. N., Owolabi R. S., Braimoh R. W. (2013). Prevalence of hypertension among urban slum dwellers in Lagos, Nigeria. *Journal of Health*.

[6] Van De Vijver S. J. M., Oti S. O., Agyemang C., Gomez G. B., Kyobutungi C. (2013). Prevalence, awareness, treatment and control of hypertension among slum dwellers in Nairobi, Kenya. *Journal of Hypertension*.

[7] Mbanya J. C. N., Minkoulou E. M., Salah J. N., Balkau B. (1998). The prevalence of hypertension in rural and urban Cameroon. *International Journal of Epidemiology*.

[8] Atashili J. Epidemiology of hypertension in the Fako Division, Cameroon (Hypertension in the Fako Division, Cameroon: Prevalence and risk factors).

[9] Dzudie A., Kengne A. P., Muna W. F. T. (2012). Awareness, treatment, and control of Hypertension. *BMJ Open*.

[10] Kingue S., NdongNgoe C., Menanga A., Fesuh B., Nouedoui C., Muna W. F. T. (2015). Prevalence and risk factors of hypertension in urban areas of cameroon: a nationwide population-based cross-sectional study. *The Journal of Clinical Hypertension*.

[11] Kamadjeu R. M., Edwards R., Atanga J. S., Unwin N., Kiawi E. C., Mbanya J.-C. (2006). Prevalence, awareness and management of hypertension in Cameroon: findings of the 2003 Cameroon Burden of Diabetes Baseline Survey. *Journal of Human Hypertension*.

[12] Ramirez S., Enquobahrie D., Nyadzi G. (2010). Prevalence and correlates of hypertension in rural Africa. *Journal of Human Hypertension*.

[13] Chobanian A. V., Bakris G. L., Black H. R. (2003). Seventh report of the joint national committee on prevention, detection, evaluation, and treatment of high blood pressure. *Hypertension*.

[14] WHO http://www.who.int/chp/steps/en/.

[15] Amoah A. G. B. (2003). Hypertension in Ghana: a cross-sectional community prevalence study in Greater Accra. *Ethnicity and Disease*.

[16] Adebayo R. A., Balogun M. O., Adedoyin R. A., Obashoro-John O. A., Bisiriyu L. A., Abiodun O. O. (2013). Prevalence of hypertension in three rural communities of Ife North Local Government Area of Osun State, South West Nigeria. *International Journal of General Medicine*.

[17] Ratovoson R., Rasetarinera O. R., Andrianantenaina I., Rogier C., Piola P., Pacaud P. (2015). Hypertension, a neglected disease in rural and urban areas in Moramanga, Madagascar. *PLoS ONE*.

[18] Williams E. A., Keenan K. E., Ansong D. (2013). The burden and correlates of hypertension in rural Ghana: a cross-sectional study. *Diabetes & Metabolic Syndrome*.

[19] Chow C. K., Teo K. K., Rangarajan S. (2013). Prevalence, awareness, treatment, and control of hypertension in rural and urban communities in high-, middle-, and low-income countries. *Journal of the American Medical Association*.

[20] Addo J., Amoah A. G. B., Kwadwo K. A. (2006). The changing patterns of hypertension in Ghana: a study of four rural communities in the Ga District. *Ethnicity and Disease*.

[21] Chataut J., Adhikari R. K., Sinha N. P. (2011). The prevalence of and risk factors for hypertension in adults living in central development region of Nepal. *Kathmandu University Medical Journal*.

[22] Ismail I. M., Kulkarni A. G., Meundi A. D., Amruth M. (2016). A community-based comparative study of prevalence and risk factors of hypertension among urban and rural populations in a coastal town of South India. *Sifa Medical Journal*.

[23] Kishore J., Gupta N., Kohli C., Kumar N. (2016). Prevalence of hypertension and determination of its risk factors in rural Delhi. *International Journal of Hypertension*.

[24] Weber M. A., Neutel J. M., Cheung D. G. (1989). Hypertension in the aged: a pathophysiologic basis for treatment. *The American Journal of Cardiology*.

[25] Cheung B. M. Y., Li C. (2012). Diabetes and hypertension: is there a common metabolic pathway?. *Current Atherosclerosis Reports*.

[26] Hendriks M. E., Wit F. W. N. M., Roos M. T. L. (2012). Hypertension in Sub-Saharan Africa: cross-sectional surveys in four rural and urban communities. *PLoS ONE*.

[27] Alikor C. A., Emem-Chioma P. C., Odia O. J. (2013). Hypertension in a rural community in River States, Niger Delta Region of Nigeria: prevalence and risk factors. *The Nigerian Health Journal*.

[28] Thawornchaisit P., De Looze F., Reid M. C., Seubsman S., Sleigh A. C. (2013). Health risk factors and the incidence of Hypertension: 4-year prospective findings from a national cohort of 60,569 Thai Open University Students. *BMJ Open*.

[29] Yuvaraj B. Y., Nagendra Gowda M. R., Umakantha A. G. (2010). Prevalence, awareness, treatment, and control of hypertension in rural areas of Davanagere. *Indian Journal of Community Medicine*.

[30] Barqora S., Campos-Nondo I., Barrera L. H. (2010). Hypertension in Mexican adults: results from the natural health and nutritional survey. *Salud Pública de México*.

[31] Bansal S. K., Saxena V., Kandpal S. D., Gray W. K., Walker R. W., Goel D. (2012). The prevalence of hypertension and hypertension risk factors in a rural Indian community: a prospective door-to-door study. *Journal of Cardiovascular Disease Research*.

